# The effect of basic fibroblast growth factor on regeneration in a surgical wound model of rat submandibular glands

**DOI:** 10.1038/ijos.2015.36

**Published:** 2015-11-20

**Authors:** Fumitaka Kobayashi, Kenichi Matsuzaka, Takashi Inoue

**Affiliations:** Department of Clinical Pathophysiology, Tokyo Dental College, Tokyo, Japan

**Keywords:** animal model, basic fibroblast growth factor, collagen, immunohistochemistry, salivary gland, wound healing

## Abstract

This study developed an animal model of surgically wounded submandibular glands (SMGs) and investigated the effects of collagen gel with basic fibroblast growth factor (bFGF) on tissue regeneration of surgically wounded SMGs *in vivo*. The animal model was produced by creating a surgical wound using a 3-mm diameter biopsy punch in SMGs. The wound was filled with collagen gel with bFGF (bFGF group) or without bFGF (control group). In the animal model of surgically wounded SMGs, salivary glands without scar tissue around the wound area were observed with smaller areas of collagen gel. Small round and spindle-shape cells invaded the collagen gel in both groups after operation day (AOD) 5, and this invasion dramatically increased at AOD 7. Host tissue completely replaced the collagen gel at AOD 21. The invading immune cells in the group treated with collagen gel with bFGF were positive for vimentin, α-smooth muscle actin (αSMA), CD49f, c-kit and AQP5 at AOD 7. Similarly, the mRNA expression of vimentin, αSMA, CD49f, keratin19 and AQP5 was also increased. This study suggests that the use of collagen gels with bFGF improves salivary gland regeneration.

## Introduction

Glandular tissues, including salivary glands, exhibit weak regenerative capacity at the site of tissue damage because these glands are composed of well-differentiated epithelial cells.^1,2,3^ Damage to salivary glands results in hyposalivation, which disturbs pronunciation and swallowing abilities and hinders lubrication function and enzymatic digestion.^4,5,6^ Saliva contains antibacterial agents and antibodies that prevent microbial proliferation in the oral cavity and play a key role in maintaining homeostasis in the oral cavity, such as pH maintenance, due to its buffering action.^4,5,6,7,8^ The loss of these functions significantly affects the patient's quality of life.^1,2,3^ Therefore, the identification of strategies to regenerate damaged salivary gland tissues is an important challenge.

Some animal models of wound healing in submandibular glands (SMGs) have been developed, such as duct ligation, radiation and surgical wounding. The wound in the surgical wound model is created by the cutting of parenchymal tissue, which alters the cellular composition in the wound area to include surrounding connective tissues rather than SMGs cells.^9,10^ Primary wound healing starts with a vascular response followed by cell migration into the wounded area. Migration is followed by inflammatory cell infiltration and degenerative changes in acinar cells.^9,10^ Subsequently, cells in intercalated ducts, which are generally positive for stem cell markers and myoepithelial cell markers, begin to proliferate and differentiate into acinar cells.^9,10^ However, secondary healing in salivary gland tissues generally includes scar tissue formation without the regeneration of salivary gland tissue.^11^ Surgical treatment of salivary glands sometimes results in secondary healing and functional disorder. We hypothesised that a method to increase fibroblast infiltration from the surrounding connective tissue into the wound area to accelerate progenitor cell proliferation and differentiation is needed to generate regenerative tissue instead of scar tissue. Therefore, tissue engineering techniques, including cells, scaffolds and growth factors, should be used.

Type I collagen gel is used as a biological scaffold.^12^ Collagen gels play an important role in cellular proliferation and differentiation *in vitro*^13,14,15,16,17^ and *in vivo*.^18^ Collagen gels facilitate wound healing^19^ and the production of luminal structural formation of the salivary gland.^20^

Basic fibroblast growth factor (bFGF) is a well-known mitogen that influences chemotaxis, cell differentiation, proliferation and tissue regeneration in general. bFGF is often used clinically and it plays a role in the wound healing^21^ of cartilage,^22^ skin,^23^ cornea,^24^ eardrum^25^ and salivary glands.^26,27,28^ bFGF also affects regeneration in periodontal^29^ and bone tissues.^30^

Some reports of wound healing of salivary glands have used wound models, such as radiation, ductal ligation and surgical wounding, but there has been no report of a surgical wound model for secondary wound healing using tissue engineering techniques.

This study created a surgically wounded model to investigate the tissue regeneration of severely wounded salivary glands using bFGF in a collagen gel *in vivo*.

## Materials and methods

### Animals

This study was conducted in compliance with the Guidelines for the Treatment of Experimental Animals at the Tokyo Dental College (Approval Number 243208). Thirty adult male Sprague–Dawley rats, each weighing approximately 200 g (Sankyo Lab Service, Tokyo, Japan), were used in this study. No animals became infected or died during the experimental period.

### Preparation of collagen gels

An acid-soluble type 1 collagen solution (3 mg·mL^−1^, pH 3) derived from porcine tendon (Cellmatrix Type I-A; Nitta Gelatin, Osaka, Japan) yielded transparent gels with consistently high gel strength after reconstitution for use in this study.

### Preparation of bFGF in collagen gel

Recombinant bFGF powder (50 µg) (Pepro Tech, Rocky Hill, NJ, USA) was dissolved in 1 mL phosphate-buffered saline (PBS) to create the bFGF stock solution (50 µg·mL^−1^). This bFGF solution was diluted 100-fold in the collagen gel solution described above to a final concentration of 500 µg·mL^−1^ bFGF in the collagen gel.^31^

### Wound model in SMGs

**Figure 1:**
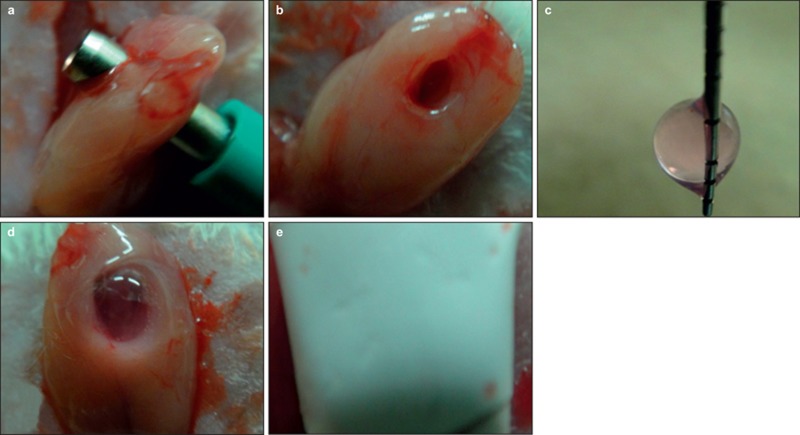
**The method for surgical wound model of SMGs.** Cells in expressed wound area were collected by collagen gel. Furthermore, we made collagen gel contained bFGF, and observed effect to these cells using this collagen gel. (**a, b**) A cylindrical defect, 3 mm in diameter, passing through the SMGs, was made in each salivary gland using a biopsy punch. (**c, d**) Collagen gels were placed in the wounds after bleeding was arrested. (**e**) Salivary glands were covered with a Gore-Tex membrane to prevent the migration of fibroblasts from the connective tissue and entry into the collagen gels. bFGF, basic fibroblast growth factor; SMG, submandibular gland.

Rats were anesthetised by intraperitoneal injection of thiopental (0.2 mL/100 g; Ravonal; Mitsubishi Tanabe, Osaka, Japan). A skin incision, approximately 2 cm in length, was made along the centre of the anterior neck using a surgical knife. Both sides of the SMGs were exposed, and a wound that passed through the SMGs was created using a 3-mm diameter biopsy punch (Kai Industries, Gifu, Japan), without injuring the principal artery and main duct (Figure 1a and 1b). Bleeding was arrested with gauze, and collagen gels with bFGF (bFGF group) or without bFGF (control group) were inserted into the wounds (Figure 1c and 1d). Wounds with collagen gels were covered with a Gore-Tex membrane (GORE, Tokyo, Japan) to prevent the migration of surrounding fibroblasts into the collagen gel (Figure 1e). The skin incision was sutured using a 4–0 non-absorbable suture. We also included an untreated control group in which the wound was not treated with collagen gel, bFGF or Gore-Tex membrane (N group).

### Histological observations

Two rats in each group were euthanised on after operation day (AOD) 5, 7, 10, 14 and 21 (*n* = 20). Rats in the N group were euthanised only on AOD 14 (*n* = 2). Animals were anesthetised by intraperitoneal injection of thiopental (Ravonal; Mitsubishi Tanabe, Osaka, Japan), and perfusion fixation was performed by transcardial injection of 10% neutral buffered formalin for 1 hour. SMGs were removed by mechanical dissection and immersed in the same fixative solution for 24 hours at room temperature. Specimens were dehydrated in ethanol before being embedded in paraffin. Paraffin sections, 4 μm in thickness, were cut horizontally using a sliding microtome. Paraffin sections were stained with haematoxylin and eosin (HE) for light microscopy observations.

### Measurement of collagen gel volume

The area of collagen gel in the specimens was measured using an Axio microscope system in each group at each of the time periods (mm^2^).

### Immunohistochemical observations

Paraffin sections of specimens at AOD 5 and 7 were used for immunohistochemical observations. Sections were deparaffinised with xylol and microwaved in a 0.01 mol·L^−1^ citric acid buffer solution (pH 6.0) for 15 min at 65 °C for antigen retrieval. Sections were incubated in 3% hydrogen peroxide with methanol for 30 min at room temperature to block endogenous peroxidase activity. The sections were treated with 3% bovine serum albumin for 10 min at room temperature to block non-specific binding. The monoclonal antibody supplied in the kit was used as the primary antibody. The sections were incubated at 4 °C overnight and incubated with a biotinylated secondary antibody, NICHIREI-Histofine simple-stain MAX-PO (Nichirei, Tokyo, Japan), for 30 min at room temperature. The sections were rinsed with PBS and stained with NICHIREI-Histofine simple-stain Diaminobenzidine (Nichirei, Tokyo, Japan) and counterstained with haematoxylin. Specimens were observed by light microscopy (Axio-photo 2; Carl Zeiss, Oberkochen, Germany). Antibodies to vimentin (Dako, Glostrup, Denmark; diluted 1:100) as a fibroblast marker, α-smooth muscle actin (αSMA; Santa Cruz Biotechnology, Dallas, TX, USA; diluted 1:50) as a muscle marker, Pan-cytokeratin (Pan-CK; Abcam, Cambridge, UK; diluted 1:40) as an epithelial cell marker, CD49f (Santa Cruz Biotechnology, Dallas, Tx, USA; diluted 1:50) as a progenitor cell marker, c-kit (Santa Cruz Biotechnology, Dallas, Tx, USA; diluted 1:50) as a progenitor cell marker, and Aquaporin 5 (AQP5; Abcam, Cambridge, UK; diluted 1:500) as an acinar cell marker, were used as primary antibodies. Cells immunopositive for these antibodies in the collagen gel of each group were counted using an Axio microscope system.

### Quantitative reverse transcription polymerase chain reaction

Total RNA was extracted using the acid guanidinium thiocyanate/phenol chloroform method as follows. Four rats from each group were sacrificed at AOD 7, and the SMGs were mechanically removed (*n* = 8). The collagen gels were washed with PBS and mechanically removed from the salivary glands using a stereoscope. The collagen gels were homogenised in 500 mL TRIzol Reagent (Invitrogen, Carlsbad, CA, USA) according to manufacturer's instructions, and total RNA was reverse-transcribed to complementary DNA (cDNA) using a QuantiTect Reverse Transcription Kit (Qiagen, Germantown, MD, USA). Quantitative reverse transcription polymerase chain reaction (RT-PCR) was performed using TaqMan Gene Expression Assays (Applied Biosystems; Life Technologies, Carlsbad, CA, USA) for the following target genes: vimentin, αSMA, keratin 13 (expression in the differentiated epithelium, such as ductal cells), CD49f (expression in the progenitor cells), keratin19 (expression in the undifferentiated epithelium, such as basal cells and epithelial stem cells), AQP5 and GAPDH (endogenous control) using the primers shown in Table 1. All PCRs were performed using a real-time PCR 7500 fast system (Applied Biosystems; Life Technologies, Carlsbad, CA, USA). mRNA expression levels of genes of interest were normalised against GAPDH expression, and results are designated as an expression coefficient.

### Statistical analysis

Quantitative data are presented as the means ± standard deviation and were analysed using Mann–Whitney's U test in an MS Excel 2008 add-in. Differences with a *P* value <0.05 were considered statistically significant.

## Results

### The wounds of the animal model healed salivary gland tissue without scar tissue formation

**Figure 2:**
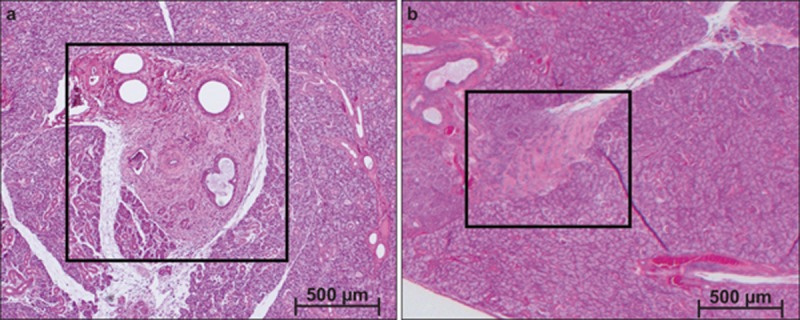
**Haematoxylin and eosin staining of submandibular glands 14 days after wound creation**. Original magnification ×25. (**a**) Wound repair without collagen gel and Gore-Tex membrane; (**b**) with collagen gel and Gore-Tex membrane.

Extensive connective tissues and healed scar tissue were observed in the wound area in the N group. A smaller collagen gel, which included cells and salivary gland tissue without scar tissue around the wound area, was observed in the control group (Figure 2a and 2b).

### bFGF accelerated wound healing

**Figure 3:**
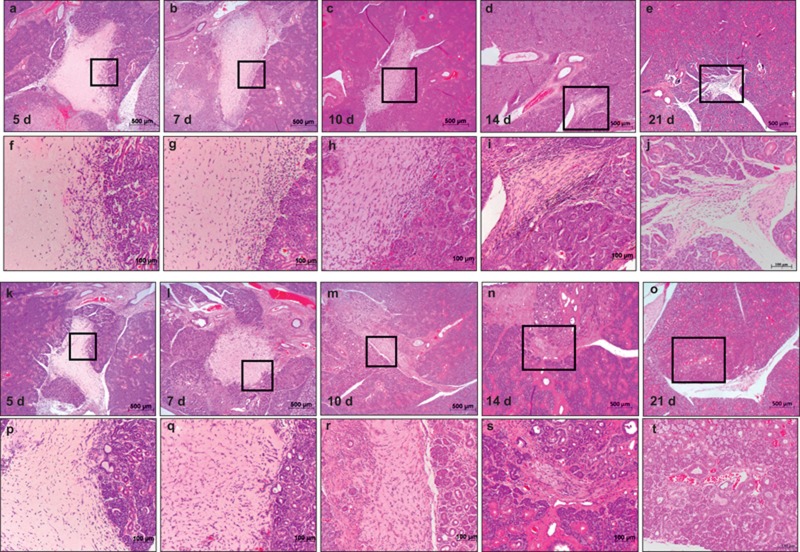
**Haematoxylin and eosin staining of submandibular glands after wound creation.** Original magnification ×25, ×100. (**a-j**) Collagen gel; (**k-t**) collagen gel with bFGF. At AOD 5, AOD 7, AOD 10 and AOD 14, the collagen gel was clearly reduced in size with time. AOD, after operation day; bFGF, basic fibroblast growth factor.

**Figure 4:**
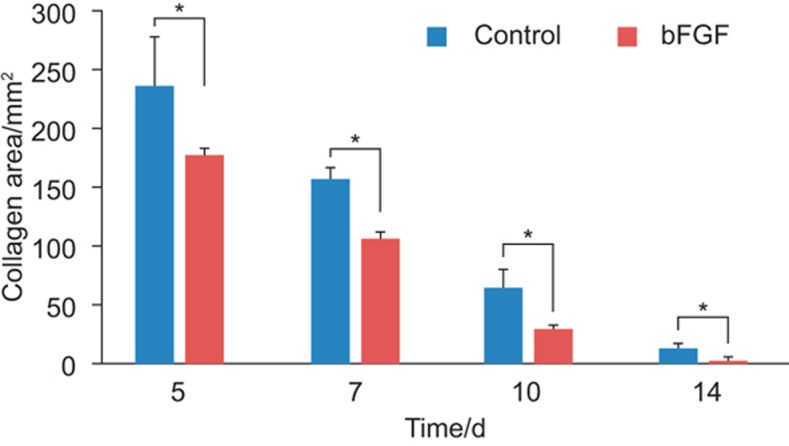
**The area of collagen gel applied to the wound site.** The area of collagen gel applied to the wound site decreased over time. The bFGF group demonstrated a significantly faster disappearance of the collagen gel area compared to the control. Data represent means ± standard deviation. *P < 0.05. bFGF, basic fibroblast growth factor.

The collagen gel was in its original state in the wound area in the low power field of both groups at AOD 5 (Figure 3a and 3k). Numerous cells were observed at AOD 5–7 (Figure 3b and 3l), which indicates increased cell infiltration. Large numbers of cells had infiltrated into the collagen gel at AOD 10 (Figure 3c and 3m), and the collagen gel and host tissue could not be distinguished clearly. Significant absorption of the collagen gel was observed from AOD 14 onward, and the collagen gel was completely replaced by host tissue at AOD 21 (Figure 3e, 3o, 3j, and 3t). Salivary gland tissue was not observed in the collagen gel during this period, despite gel absorption and replacement by host tissue. The volume of the collagen gel in the wounds of each group became progressively smaller, but collagen gels in the bFGF group were significantly smaller compared to the control group at each of the time periods (Figures 3 and 4).

High-power magnification revealed oval-shaped cells and spindle-shaped cells near the edge of the wounded salivary glands in the collagen gel at AOD 5 in the control group (Figure 3a and 3f). The number of spindle-shaped cells increased in the collagen gel at AOD 7, especially in the inner area of the collagen gel (Figure 3b and 3g). The numbers of oval-shaped and spindle-shaped cells in the collagen gel increased at AOD 10. There was a high density of these cells in the collagen gel (Figure 3c and 3h). A decrease in the size of the collagen gel was also clearly observed. The remaining collagen gel was quite small at AOD 14 (Figure 3d and 3i).

High-power magnification revealed many more cells in the collagen gel of the bFGF groups compared to the control group at AOD 5 (Figure 3k and 3p). Many of the cells in the collagen gel were spindle-shaped cells. Cells had infiltrated into the collagen gel uniformly at AOD 7 (Figure 3l and 3q). Aggregated cells in the collagen gel and the area between the collagen gel and salivary glands tissue could not be seen clearly at AOD 10 (Figure 3m and 3r). The collagen gel had almost disappeared and was replaced with host tissue at AOD 14 (Figure 3n and 3s).

### Characterisation of cell types in the wound area

#### Myoepithelial cells

**Figure 5:**
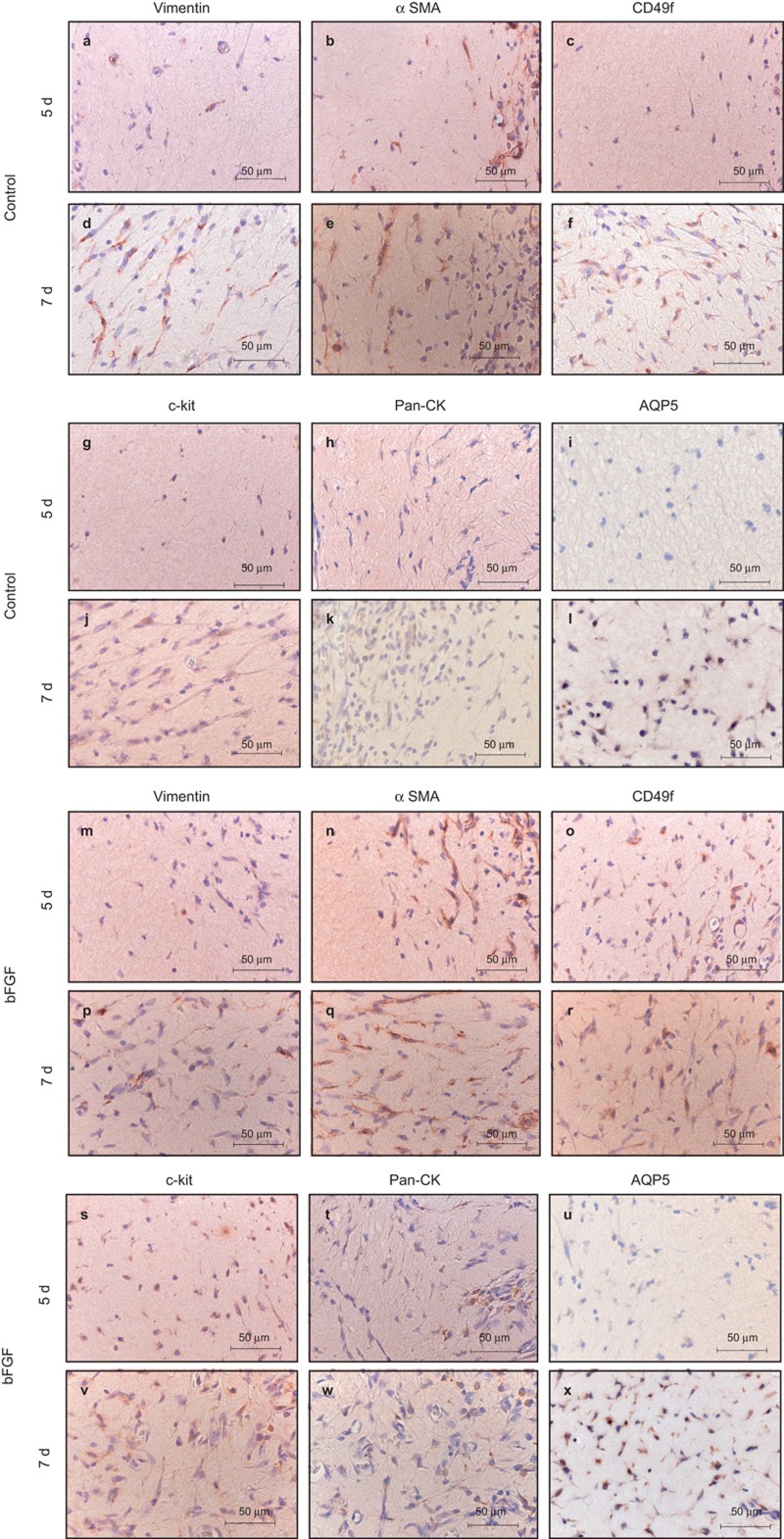
**Immunohistochemical staining.** Original magnification ×400. (**a-l**) Collagen gel; (**m-x**) collagen gel with bFGF. Collagen gel stained with antibodies to vimentin, αSMA, Pan-CK, CD49f, c-kit and AQP5 at AOD 5 and AOD 7. In the control group, vimentin- and αSMA-positive cells were observed in the peripheral area of the collagen gel at AOD 5. Pan-CK-, CD49f-, c-kit- and AQP5-positive cells were not observed. In the bFGF group, CD49f- and c-kit-positive cells were observed at AOD 5, but AQP5-positive cells were not observed. In the both groups, αSMA-positive cells were observed in the inner area of the collagen gel at AOD 7. Vimentin-, Pan-CK-, CD49f-, c-kit- and AQP5-positive cells were observed in peripheral areas of the collagen gel. AOD after operation day; AQP5, Aquaporin 5; bFGF, basic fibroblast growth factor; αSMA, α-smooth muscle actin.

Spindle-shaped cells located in the peripheral area of the collagen gel at AOD 5 were positive for vimentin (Figure 5a) and αSMA (Figure 5e).

Numerous spindle-shaped cells were positive for vimentin at AOD 7 (Figure 5d), and αSMA-positive cells were observed in the peripheral area and entire area of the collagen gel at AOD 7 (Figure 5e). Cell spreading of these cells was also observed.

#### Stem cells

Cells positive for CD49f (Figure 5c) and c-kit (Figure 5g) were not observed at AOD 5, but oval-shaped cells and a few spindle-shaped cells stained positive for CD49f (Figure 5f) and c-kit (Figure 5j) at AOD 7. Cells positive for CD49f (Figure 5o) and c-kit (Figure 5s) were seen in the bFGF group at AOD 5. These cells increased and expanded at AOD 7 (Figure 5r and 5v).

#### Ductal epithelial cells

Cells positive for Pan-CK were not observed at AOD 5 in the control group (Figure 5h), but oval-shaped Pan-CK positive cells were observed in the bFGF group (Figure 5t). Pan-CK positive cells increased slightly at AOD 7 (Figure 5k and 5w).

#### Acinar cells

Neither group exhibited AQP5-positive cells at AOD 5 (Figure 5i and 5u). However, numerous small AQP5-positive cells were observed in the peripheral area of the collagen gel at AOD 7 (Figure 5l and 5x).

### Effect of bFGF on cell proliferation and differentiation

**Figure 6:**
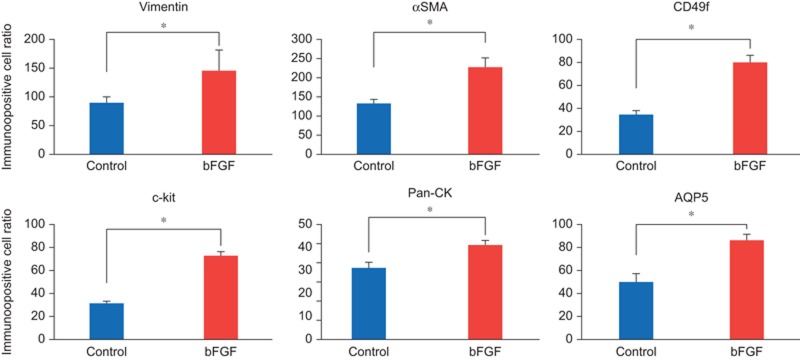
**Immunopositive cell ratios.** Vimentin, αSMA, CD49f, c-kit and AQP5 exhibited significantly higher ratios in the group with bFGF compared to the control group at AOD 7. Data represent means ± standard deviation. *P < 0.05. AOD, after operation day; AQP5, Aquaporin 5; bFGF, basic fibroblast growth factor; αSMA, α-smooth muscle actin.

Immunohistochemical observations at AOD 7 revealed that the ratios of positive cells for vimentin, αSMA, CD49f, c-kit, pan-CK and AQP5 were significantly higher in the bFGF group compared to the control group (Figure 6).

**Figure 7:**
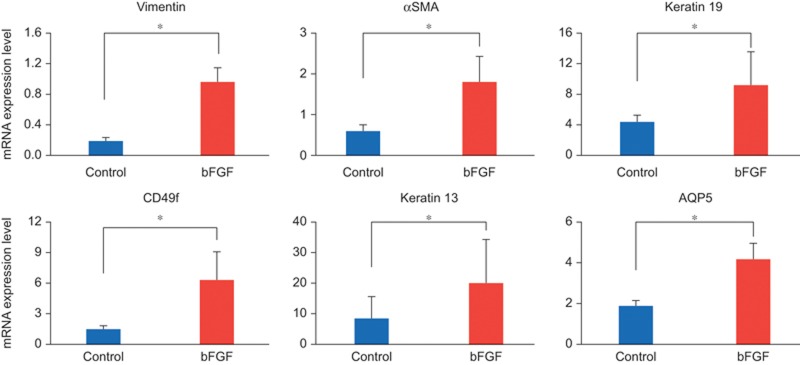
**mRNA expression levels.** mRNA analyses indicated that the expression of vimentin, αSMA, CD49f, keratin 19, keratin 13 and AQP5 was significantly higher in the group with bFGF compared to the control group at AOD 7. Data represent means ± standard deviation. *P < 0.05. AOD, after operation day; AQP5, Aquaporin 5; bFGF, basic fibroblast growth factor; αSMA, α-smooth muscle actin.

The mRNA expression levels of vimentin, αSMA, CD49f, keratin 19, keratin 13 and AQP5 were also significantly higher in the bFGF group compared to the control group at AOD 7 (Figure 7).

## Discussion

Partial resection of rat salivary glands stimulates wound healing *via* the proliferation of granulation tissue, which results in scar tissue formation over time without regenerative tissue. Rats in the N group demonstrated healing with scar tissue formation in this study. SMG damage in the wound model (control group), which we created using collagen gel and Gore-Tex membrane, was healed by cells that migrated from the salivary gland tissue into the collagen gel without scar tissue formation. Collagen gels are suitable scaffolds for cell proliferation and differentiation.^13,14,15,16,17,18,19^ Primary culture of salivary gland tissue on collagen gels has demonstrated that the collagen gel inhibits fibroblast proliferation, but it promotes the three-dimensional proliferation of duct epithelial cells and the eventual formation of the luminal structure by myoepithelial cells.^32^ Moreover, a Gore-Tex membrane was used to avoid the invasion of fibroblasts from the surrounding connective tissue. On this basis, the current surgical wound model was appropriate for observation of salivary gland wound healing.

Intercalated ducts, striated ducts, excretory ducts, myoepithelial cells and stem cells, except acinar cells, should be engaged during the wound healing of salivary glands.^33^ Acinar cells are particularly necessary for the regeneration of salivary glands. AQP5 is expressed in the acinar cells and along the intercalated ducts in SMGs,^34^ and it is important in saliva secretion for water movement across the plasma membrane.^35^ The reported reduction in AQP5 expression in the ligated gland is consistent with the defective fluid secretion of atrophic glands, but AQP5 expression recovered with salivary gland regeneration. Therefore, AQP5 may be used as an index of salivary gland regeneration after injury.^36^

CD49f and c-kit are well-known progenitor cell markers of the salivary gland. Cells positive for these markers increase during the regeneration of damaged salivary glands.^37,38^ Furthermore, these cells are pluripotent.^39,40^ Cells expressing these progenitor cell markers are located in the regenerating salivary gland, and cells that are positive for these markers may be related to the regeneration of the salivary gland. Cells positive for CD49f also express keratin19,^41^ and it has been suggested previously that cells expressing keratin19 include progenitor cells.

The results of the immunohistochemical study and RT-PCR analysis demonstrated that the numbers of cells positive for Pan-CK, α-SMA, CD49f and c-kit were much higher in the bFGF group than the control group. These results suggest that bFGF in the collagen gel promoted the proliferation of ductal cells, myoepithelial cells, fibroblasts and stem cells and helped maintain the characteristics of progenitor cells. Furthermore, AQP5-positive cells were observed in the collagen gel at AOD 7, and this result suggests that regenerated intercalated duct cells differentiate into acinar cells.

Wound healing began with an infiltration of primarily αSMA-expressing cells into the collagen gel in the current study. This infiltration was followed by the infiltration or proliferation of cells expressing vimentin, c-kit and CD49f. AQP5-expressing cells appeared last. Myoepithelial cells were observed earlier than other types of cells. An inflammatory reaction occurred first in a salivary gland regeneration model using duct ligation, which was followed by the atrophy and apoptosis of acinar cells. Myoepithelial cells and progenitor cells undergo proliferation.^42^ Regenerated intercalated duct cells originating from stem cells differentiated into acinar cells after release of the ligation, and myoepithelial cells were eventually located around the regenerated acinus. Therefore, myoepithelial cells are related to salivary gland regeneration. Myoepithelial cells produce laminin.^43,44^ Stem cells, which are positive for CD49f and c-kit, also express a high-affinity receptor for laminin.^45^ Together, our results suggest that proliferating myoepithelial cells produced laminin that bound to the stem cells at the start of wound healing in this study, and these stem cells possibly differentiate into the ductal epithelium.

bFGF also exerts effects on fibroblasts, vascular endothelial cells and epithelial cells, which results in the promotion of tissue regeneration.^46^ bFGF also promotes the proliferation of myoepithelial and ductal cells of salivary glands.^47^ Furthermore, bFGF helps in the functional maintenance of progenitor cells.^28,46^

We created a salivary gland surgical wound model that is suitable for observations of the wound healing process. Collagen gel scaffolds induce the proliferation of ductal cells, myoepithelial cells and progenitor cells, but not their differentiation to acinar cells. However, AQP5-positive cells were observed in collagen gels that included bFGF in this study, which suggests the occurrence of acinar cell regeneration.

## Conclusion

This study suggests that the use of collagen gels with bFGF for the repair of salivary gland injury improves the potential for salivary gland regeneration.

## Figures and Tables

**Table 1:** Primers used for real-time reverse transcription-polymerase chain reaction

Primer	Gene name	Assay ID
Cytokeratin 13	keratin 13	Rn01464229_ml
Cytokeratin 19	keratin 19	Rn01496867_ml
CD49f	integrin alpha6	Rn01512708_ml
αSMA	smooth muscle alpha-actin	Rn01759928_gl
Vimentin	vimentin	Rn00579738_ml
Aquaporin5	aquaporin5	Rn00562837_ml
GADPH (endogenous control)	glyceraldehyde-3-phosphate dehydrogenase	Rn01775763_gl

## References

[bib1] Atkinson JC, Fox PC. Salivary gland dysfunction. Clin Geriatr Med 1992; 8(3): 499–511. 1504941

[bib2] Fox PC. Acquired salivary dysfunction. Drugs and radiation. Ann N Y Acad Sci 1998; 842: 132–137. 9599303 10.1111/j.1749-6632.1998.tb09641.x

[bib3] Ship JA, Pillemer SR, Baum BJ. Xerostomia and the geriatric patient. J Am Geriatr Soc 2002; 50(3): 535–543. 11943053 10.1046/j.1532-5415.2002.50123.x

[bib4] Magee DF. Salivary gland. Physiology and biophysics. Philadelphia, London: W B Saunders, 1965.

[bib5] Carlson E, Ord R. Textbook and color atlas of salivary gland pathology: diagnosis and management. Hoboken: Wiley-Blackwell, 2008.

[bib6] Provenza DV. Oral histology inheritance and development. Philadelphia, Montreal: Lippincott, 1964.

[bib7] Vissink A, Burlage FR, Spijkervet FK et al. Prevention and treatment of the consequences of head and neck radiotherapy. Crit Rev Oral Biol Med 2003; 14(3): 213–225. 12799324 10.1177/154411130301400306

[bib8] Vissink A, Jansma J, Spijkervet FK et al. Oral sequelae of head and neck radiotherapy. Crit Rev Oral Biol Med 2003; 14(3): 199–212. 12799323 10.1177/154411130301400305

[bib9] Takahashi S, Schoch E, Walker NI. Origin of acinar cell regeneration after atrophy of the rat parotid induced by duct obstruction. Int J Exp Pathol 1998; 79(5): 293–301. 10193312 10.1046/j.1365-2613.1998.710405.x PMC3220221

[bib10] Man YG, Ball WD, Marchetti L et al. Contributions of intercalated duct cells to the normal parenchyma of submandibular glands of adult rats. Anat Rec 2001; 263(2): 202–214. 11360236 10.1002/ar.1098

[bib11] Matsubara H, Kajiyama M. [An experimental study of wound healing after partial extirpation of Wistar rat submandibular gland.] Kyushu-Shika-Gakkai-zasshi 1992; 46(6): 818–831. Japanese.

[bib12] Parry DAD, Creamer LK. Fibrous proteins: scientific, industrial and medical aspects. 2nd edn. Amsterdam: Academic Press, 1980.

[bib13] Gospodarowicz D, Greenburg G, Birdwell CR. Determination of cellular shape by the extracellular matrix and its correlation with the control of cellular growth. Cancer Res 1978; 38(11 Pt 2): 4155–4171. 359133

[bib14] Wicha MS, Liotta LA, Garbisa S et al. Basement membrane collagen requirements for attachment and growth of mammary epithelium. Exp Cell Res 1979; 124(1): 181–190. 499382 10.1016/0014-4827(79)90268-4

[bib15] Murray JC, Stingl G, Kleinman HK et al. Epidermal cells adhere preferentially to type IV (basement membrane) collagen. J Cell Biol 1979; 80(1): 197–202. 422650 10.1083/jcb.80.1.197 PMC2110292

[bib16] Sattler CA, Michalopoulos G, Sattler GL et al. Ultrastructure of adult rat hepatocytes cultured on floating collagen membranes. Cancer Res 1978; 38(6): 1539–1549. 565678

[bib17] Auger FA, Rouabhia M, Goulet F et al. Tissue-engineered human skin substitutes developed from collagen-populated hydrated gels: clinical and fundamental applications. Med Biol Eng Comput 1998; 36(6): 801–812. 10367474 10.1007/BF02518887

[bib18] Yang W, Both SK, van Osch GJ et al. Performance of different three-dimensional scaffolds for *in vivo* endochondral bone generation. Eur Cell Mater 2014; 27: 350–364. 24913441 10.22203/ecm.v027a25

[bib19] Bornstein P, Traub W. The chemistry and biology of collagen// Neurath H, Hill R eds. The proteins. 3rd edn. Amsterdam: Academic Press, 1979: 411–632.

[bib20] Yoneda T, Imamoto A, Sakuda M. [Primary culture of mouse submandibular gland epithelial cells embedded in collagen gel matrix.] Jpn J Oral Biol 1986; 28: 12–18. Japanese.

[bib21] Rifkin DB, Moscatelli D. Recent developments in the cell biology of basic fibroblast growth factor. J Cell Biol 1989; 109(1): 1–6. 2545723 10.1083/jcb.109.1.1 PMC2115467

[bib22] Cuevas P, Burgos J, Baird A. Basic fibroblast growth factor (FGF) promotes cartilage repair *in vivo*. Biochem Biophys Res Commun 1988; 156(2): 611–618. 3190672 10.1016/s0006-291x(88)80887-8

[bib23] Mazué G, Bertolero F, Jacob C et al. Preclinical and clinical studies with recombinant human basic fibroblast growth factor. Ann N Y Acad Sci 1991; 638: 329–340. 1785810 10.1111/j.1749-6632.1991.tb49043.x

[bib24] Hoppenreijs VP, Pels E, Vrensen GF et al. Basic fibroblast growth factor stimulates corneal endothelial cell growth and endothelial wound healing of human corneas. Invest Ophthalmol Vis Sci 1994; 35(3): 931–944. 8125756

[bib25] Fina M, Baird A, Ryan A. Direct application of basic fibroblast growth factor improves tympanic membrane perforation healing. Laryngoscope 1993; 103(7): 804–809. 8341107 10.1288/00005537-199307000-00015

[bib26] Thula TT, Schultz G, Tran-Son-Tay R et al. Effects of EGF and bFGF on irradiated parotid glands. Ann Biomed Eng 2005; 33(5): 685–695. 15981868 10.1007/s10956-005-1853-z

[bib27] Cotrim AP, Sowers A, Mitchell JB et al. Prevention of irradiation-induced salivary hypofunction by microvessel protection in mouse salivary glands. Mol Ther 2007; 15(12): 2101–2106. 17726456 10.1038/sj.mt.6300296

[bib28] Kojima T, Kanemaru S, Hirano S et al. The protective efficacy of basic fibroblast growth factor in radiation-induced salivary gland dysfunction in mice. Laryngoscope 2011; 121(9): 1870–1875. 22024837 10.1002/lary.21873

[bib29] Ishii Y, Fujita T, Okubo N et al. Effect of basic fibroblast growth factor (FGF-2) in combination with beta tricalcium phosphate on root coverage in dog. Acta Odontol Scand 2013; 71(2): 325–332. 22545917 10.3109/00016357.2012.680906

[bib30] Furuya H, Tabata Y, Kaneko K. Bone regeneration for murine femur fracture by gelatin hydrogels incorporating basic fibroblast growth factor with different release profiles. Tissue Eng Part A 2014; 20(9/10): 1531–1541. 24410201 10.1089/ten.TEA.2012.0763

[bib31] Hiramatsu Y, Kagami H, Horie K et al. Effects of basic fibroblast growth factor on cultured rat and human submandibular salivary gland cells. Arch Oral Biol 2000; 45(7): 593–599. 10785523 10.1016/s0003-9969(99)00148-x

[bib32] Lee EY, Xia Y, Kim WS et al. Hypoxia-enhanced wound-healing function of adipose-derived stem cells: increase in stem cell proliferation and up-regulation of VEGF and bFGF. Wound Repair Regen 2009; 17(4): 540–547. 19614919 10.1111/j.1524-475X.2009.00499.x

[bib33] Ihrler S, Zietz C, Sendelhofert A et al. A morphogenetic concept of salivary duct regeneration and metaplasia. Virchows Arch 2002; 440(5): 519–526. 12021927 10.1007/s004280100537

[bib34] Matsuzaki T, Suzuki T, Koyama H et al. Aquaporin-5 (AQP5), a water channel protein, in the rat salivary and lacrimal glands: immunolocalization and effect of secretory stimulation. Cell Tissue Res 1999; 295(3): 513–521. 10022971 10.1007/s004410051257

[bib35] Ma T, Song Y, Gillespie A et al. Defective secretion of saliva in transgenic mice lacking aquaporin-5 water channels. J Biol Chem 1999; 274(29): 20071–20074. 10400615 10.1074/jbc.274.29.20071

[bib36] Cotroneo E, Proctor GB, Paterson KL et al. Early markers of regeneration following ductal ligation in rat submandibular gland. Cell Tissue Res 2008; 332(2): 227–235. 18335244 10.1007/s00441-008-0588-6 PMC2493059

[bib37] Nanduri LS, Maimets M, Pringle SA et al. Regeneration of irradiated salivary glands with stem cell marker expressing cells. Radiother Oncol 2011; 99(3): 367–372. 21719134 10.1016/j.radonc.2011.05.085

[bib38] Lombaert IM, Brunsting JF, Wierenga PK et al. Rescue of salivary gland function after stem cell transplantation in irradiated glands. PLoS One 2008; 3(4): e2063. 18446241 10.1371/journal.pone.0002063 PMC2329592

[bib39] Petrakova OS, Terskikh VV, Chernioglo ES et al. Comparative analysis reveals similarities between cultured submandibular salivary gland cells and liver progenitor cells. Springerplus 2014; 3: 183. 24790827 10.1186/2193-1801-3-183 PMC4000360

[bib40] Okumura K, Nakamura K, Hisatomi Y et al. Salivary gland progenitor cells induced by duct ligation differentiate into hepatic and pancreatic lineages. Hepatology 2003; 38(1): 104–113. 12829992 10.1053/jhep.2003.50259

[bib41] Matsumoto S, Okumura K, Ogata A et al. Isolation of tissue progenitor cells from duct-ligated salivary glands of swine. Cloning Stem Cells 2007; 9(2): 176–190. 17579551 10.1089/clo.2006.0022

[bib42] Takahashi S, Kohgo T, Nakamura S et al. Biological behavior of myoepithelial cells in the regeneration of rat atrophied sublingual glands following release from duct ligation. J Mol Histol 2005; 36(5): 373–379. 16283425 10.1007/s10735-005-9009-2

[bib43] Warburton MJ, Ormerod EJ, Monaghan P et al. Characterization of a myoepithelial cell line derived from a neonatal rat mammary gland. J Cell Biol 1981; 91(3 Pt 1): 827–836. 7199047 10.1083/jcb.91.3.827 PMC2112821

[bib44] Warburton MJ, Ferns SA, Rudland PS. Enhanced synthesis of basement membrane proteins during the differentiation of rat mammary tumour epithelial cells into myoepithelial-like cells *in vitro*. Exp Cell Res 1982; 137(2): 373–380. 7035201 10.1016/0014-4827(82)90038-6

[bib45] Aumailley M, Timpl R, Sonnenberg A. Antibody to integrin alpha 6 subunit specifically inhibits cell-binding to laminin fragment 8. Exp Cell Res 1990; 188(1): 55–60. 2139418 10.1016/0014-4827(90)90277-h

[bib46] Okumura N, Takimoto K, Okada M et al. C6 glioma cells produce basic fibroblast growth factor that can stimulate their own proliferation. J Biochem 1989; 106(5): 904–909. 2613696 10.1093/oxfordjournals.jbchem.a122950

[bib47] Haimovitz-Friedman A, Balaban N, McLoughlin M et al. Protein kinase C mediates basic fibroblast growth factor protection of endothelial cells against radiation-induced apoptosis. Cancer Res 1994; 54(10): 2591–2597. 8168085

